# Hidden in the Pangenome? Machine Learning-Driven Discovery of Antimicrobial Potential in *Corynebacterium glutamicum*

**DOI:** 10.1177/11779322261461933

**Published:** 2026-06-17

**Authors:** Sk Injamamul Islam, Khandker Shahed, Mst. Mahmuda Parvin, Saloa Sanjida

**Affiliations:** 1 BioMac Lab, Dhaka, Bangladesh; 2 Department of Fisheries Biology and Genetics, Faculty of Fisheries, Aquaculture and Marine Science, 684085 Shere-Bangla Agricultural University, Dhaka, Bangladesh

**Keywords:** machine learning, antimicrobial peptides, bacteria, biosynthetic gene, pangenome

## Abstract

Antimicrobial resistance poses an increasing global challenge, driving the urgent need for alternative strategies to identify novel therapeutic agents. Microbial natural products encoded by biosynthetic gene clusters (BGCs) remain among the most promising sources of bioactive compounds. Although *Corynebacterium glutamicum* is best known as an industrial producer of amino acids, its potential as a producer of secondary metabolites has not been comprehensively assessed, despite the availability of numerous high-quality genome sequences. In this study, we carried out a comparative pangenome analysis of 36 complete *C. glutamicum* genomes and systematically mined for BGCs to explore the species’ biosynthetic repertoire. Our analysis revealed variation in BGC content among strains, with several isolates harboring more hybrid clusters than others, suggesting metabolic diversity across the species. In addition to conserved terpene biosynthetic pathways, we detected polyketide-associated clusters not previously reported in *C. glutamicum*, expanding its recognized metabolic potential. RiPP-like clusters, including Lactococcin-related variants, were also identified, highlighting an underexplored reservoir of antimicrobials. To prioritize candidates for future validation, Support Vector Machine, Random Forest, and k-Nearest Neighbor models were trained on Composition, Transition, and Distribution (CTD) physicochemical sequence features and applied to genome-mined small open reading frames. The models demonstrated strong predictive performance, with the Support Vector Machine achieving the highest accuracy (84.1%), F1 score (83.8%), and area under the ROC curve (AUC = 0.920). After removing duplicate sequence IDs and applying a high-confidence AMP probability threshold (≥0.95), 18 unique AMP-like candidates were identified as promising. Overall, this study presents *C. glutamicum* as a promising source of bioactive metabolite candidates and shows how pangenome-scale mining combined with machine learning can support antimicrobial peptide discovery while still requiring experimental validation of the predicted leads.

## 1. Introduction

Natural products (NPs) have received significant attention due to their diverse structures and valuable applications, particularly in health, food, and agriculture.^1^ Microorganisms play a crucial role in producing NPs, serving as key contributors to drug discovery.^2,3^ The escalating emergence of resistance to existing therapeutics underscores an urgent need for the development of novel pharmacological interventions to effectively address this global challenge.^4^ As a result, alternative sources and innovative approaches are essential for uncovering novel therapeutic agents.

Numerous genome mining efforts have concentrated on well-characterized secondary metabolite producers, including bacilli, actinobacteria, and myxobacteria.^5,6^ *In silico* analysis of microbial genome sequences utilizing advanced bioinformatics methods has demonstrated that a single genome may harbor 20-80 distinct biosynthetic gene clusters (BGCs), the majority of which are cryptic and fail to express under conventional laboratory culture conditions, indicating a substantial reservoir of unexplored compounds.^7,8^ However, conventional genome mining approaches are limited in their ability to predict novel or divergent BGC architectures, as they rely primarily on homology-based or rule-driven detection methods.^9^ This limitation highlights the need for integrative computational strategies, including machine learning, to improve the identification and functional annotation of previously uncharacterized clusters.

Compared with several well-known producers of secondary metabolites, *Corynebacterium glutamicum* benefits from a greater number of completely sequenced genomes, more accurate genome annotations, well-maintained, curated databases, and a wide array of tools for data analysis.^10-12^ This bacterium is primarily known for its industrial applications, particularly in large-scale production of amino acids such as glutamate and lysine. Its genetic tractability and metabolic versatility make it a valuable model organism in biotechnology and synthetic biology.^13^

For the past five decades, industrialized nations have relied on *C. glutamicum*’s remarkable capacity to produce and excrete large quantities of amino acids.^14^ The compounds produced through *C. glutamicum* fermentation are utilized in various sectors, including animal feed, dietary supplements, cosmetics, flavor enhancers, and pharmaceutical synthesis.^15^ Compared to *Escherichia coli* and *Saccharomyces cerevisiae*,^16^ *C. glutamicum* demonstrates significant potential to outperform other industrial microorganisms and offers several advantages. Firstly, *C. glutamicum* exhibits low extracellular protease activity and can secrete properly folded, functional precursor proteins.^17^ Secondly*, C. glutamicum* exhibits minimal carbon catabolite repression, allowing it to utilize mixed sugars as a carbon source without significant growth inhibition.^18,19^ Thirdly, *C. glutamicum* is highly resilient, showing tolerance to organic acids, furfural, and toxic aromatic compounds. In addition, it retains strong catalytic activity under growth-inhibitory conditions while supporting robust cell proliferation in high-density fermentation processes.^20-22^ These characteristics are especially beneficial for managing the toxic by-products of lignocellulose processing and for degrading environmental pollutants. Fourthly, *C. glutamicum* can metabolize a wide range of natural carbon substrates, including five-carbon sugars (D-xylose and L-arabinose), six-carbon sugars (glucose and mannose), monosaccharides (maltose), and toxic aromatic compounds.^23,24^ The substrate range of *C. glutamicum* encompasses three generations of bio-refining raw materials. Additionally, *C. glutamicum* is safe, has a well-defined genetic background, can produce a variety of compounds, and can convert inexpensive biomass into valuable products.^13^ However, despite the vast amount of genomic data available for *C. glutamicum*, comprehensive identification of metabolites on a pangenome-wide scale remains underexplored. This limitation restricts the discovery of novel metabolites, as research efforts have largely focused on previously characterized pathways and compounds. Although many C. glutamicum genome sequences are available in public databases, indicating considerable untapped potential for metabolic diversity, reports of unique or novel metabolites remain scarce. This reveals a critical gap in our understanding of its biosynthetic capacity. While *C. glutamicum* has been studied for the production of antibiotics and other bioactive molecules, the occurrence of ribosomally synthesized and post-translationally modified peptide (RiPP)-like gene clusters remains poorly characterized. Given the structural diversity and therapeutic relevance of RiPPs, integrating machine learning models trained on antimicrobial peptide datasets may provide a robust framework for predicting their bioactivity and guiding experimental validation.^25^ Furthermore, comprehensive exploration of silent biosynthetic gene clusters and their metabolic products holds promise for advancing drug discovery and broadening industrial applications.^20^

**Figure 1.**
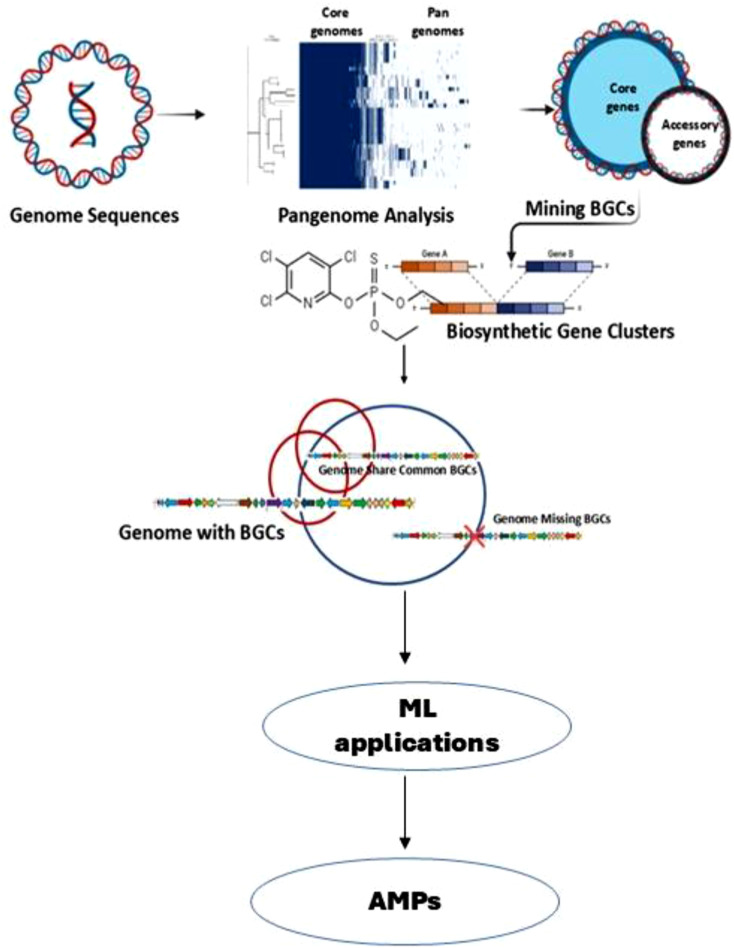
Schematic representation of the presented large-scale workflow, integrating genome mining and pangenome analysis

Recent advances in machine learning have demonstrated high accuracy in predicting antimicrobial peptides and classifying secondary metabolite biosynthetic pathways directly from sequence-derived features.^25-27^ Despite these advances, machine learning has not yet been widely integrated with pangenome analysis and BGC mining of *C. glutamicum*, leaving a gap in leveraging predictive models to uncover its antimicrobial potential. The objective of this study is to conduct a comprehensive *in silico* investigation of BGCs in *C. glutamicum* by integrating pangenome analysis, phylogenomics, and genome mining. Specifically, this work aims to (i) assess the diversity and distribution of BGCs across the *C. glutamicum* pangenome, (ii) determine the contribution of unique or strain-specific clusters to unexplored secondary metabolite diversity, and (iii) evaluate the potential of machine learning approaches to enhance the prediction and prioritization of antimicrobial-associated BGCs compared to conventional genome mining tools. Within this framework (Figure 1), the study seeks to provide new insights into *C. glutamicum*’s biosynthetic capacity and to lay a foundation for future exploration of its natural product potential.^28^

In this workflow, machine learning was used as a complementary step rather than a replacement for established genome-mining tools. AntiSMASH and BAGEL5 are valuable because they detect BGC and RiPP signatures using curated rules, sequence homology, and profile hidden Markov models. However, highly divergent AMP-like peptides may show weak or no obvious similarity to known peptide families. CTD-based machine learning can therefore add a useful prioritization layer by ranking peptides according to physicochemical properties such as charge, hydrophobicity, polarity, polarizability, solvent accessibility, van der Waals volume, and secondary-structure tendency. This allows the analysis to focus not only on known sequence similarity, but also on AMP-like biochemical properties.

## 2. Methods

Reporting guideline statement: This computational comparative genomics and machine-learning study was prepared in accordance with relevant EQUATOR reporting principles. A completed reporting checklist is submitted as supplementary material where applicable; clinical and participant-related items are marked as not applicable because the study used publicly available genome sequences and computational peptide-prediction analyses only.

### 2.1. C. glutamicum Dataset Collection and Quality Assessment

A total of 82 distinct *C. glutamicum* genome entries, including contigs, scaffolds, chromosomes, and complete sequences, were available in the NCBI genome database at the time of data collection (accessed August 3, 2024). On August 20, 2024, we downloaded 36 complete and annotated *C. glutamicum* genomes for the final pangenome and BGC analysis (Supplementary Table 1). Draft assemblies, contigs, and scaffold-level genomes were excluded because fragmented assemblies can split biosynthetic gene clusters, obscure cluster boundaries, increase apparent gene absence, and reduce comparability across strains. This filtering step was used to improve the reliability of Prokka re-annotation, Roary pangenome construction, and antiSMASH/BAGEL5-based cluster mining. Recent studies have emphasized the importance of selecting high-quality genome datasets for pangenome analysis.^29-31^

### 2.2. Genomic Comparison

Nucleotide-level comparisons of all possible genome pairs were performed using the average nucleotide identity (ANI) method to validate species relationships. Pairwise ANI values were calculated with PyANI v 0.2.12, employing two different computational methods, the MUMmer^32^ and the BLAST+ method^33^ employing the ANIm and ANIb options, respectively. All strains classified as *C. glutamicum* were chosen for further examination, whereas those misidentified were omitted. This was determined by setting a threshold value showing that the presence of the same species of ANI exceeds 94%.^34,35^

### 2.3. Pan-Genome Construction

*C. glutamicum* genomes were re-annotated using Prokka v1.14.6 to achieve standardized and consistent functional annotations.^36^ Roary^37^ with a default identity threshold of 95%, was employed to construct the core and pan-genome of *C. glutamicum*. In addition, the pangenome was analyzed using Heap’s Law^38^ and Power Law fit^39^ in conjunction with the custom script (https://github.com/SethCommichaux/Heap_Law_for_Roary) and the “ggcaller v1.3.0” in Python programming,^40^ respectively. This facilitated the calculation of constant variables and allowed the application of the least squares method to fit an exponential regression decay model to both the core genome and singleton genes. Heap’s Law formula was employed to deduce the number of genes that will constitute the core genome once it becomes stable^41^ and to provide an approximate estimation of the number of genes introduced by each newly sequenced genome.

### 2.4. Identification of BGCs and Construction of Phylogenetic Tree

AntiSMASH v7.0,^42^ was utilized to predict and annotate BGCs responsible for producing secondary metabolites in the core and pangenome of *C. glutamicum* strains. AntiSMASH detects key classes of secondary metabolite BGCs by leveraging the signature profiles of Hidden Markov Models (pHMMs) derived from multiple sequence alignments of empirically characterized proteins or protein domains.^43^ The results of unique and hybrid BGCs identified by antiSMASH are shown in Supplementary Table 2. The pHMMs employed combine those described by Medema et al.,^44^ using the same thresholds for PKS I, PKS II, PKS III, NRPS, indolocarbazoles, aerobactin-like siderophores, butyrolactones, aminoglycosides, and β-lactams, while also screening for fatty acid synthases detected by PKS models. New pHMMs were created for identifying terpene synthases based on published sequences, lanthipeptides using the essential cyclase domain, thiazole-oxazole modified microcins (TOMMs) based on the YcaO domain, and phosphonates screening sequences containing the EDK-X (5)-NS motif present in all verified PepM sequences.^45-47^ To construct a more reliable phylogeny at the BGCs level, the maximum likelihood algorithm (RAxML) was employed for comparison between the phylogeny of core biosynthetic genes.^48^ The resulting phylogenetic tree was visualized using iTOL v5 (Interactive Tree of Life)^49^ Table 1 and 2.

### 2.5. RiPPs Mining

We utilized BAGEL5, a user-friendly web server, to extensively mine for RiPPs and unmodified bacteriocins from the *C. glutamicum* genomic DNA.^50^ BAGEL5 is freely accessible at https://bagel4.molgenrug.nl. Interest in these types of molecules is growing, driven by the need for new antibiotics and antimicrobial peptides, and by their significant roles in food preservation, microbial ecology, and plant biocontrol. Our objective is to explore a wide array of topics related to this class of natural products.

### 2.6 Machine Learning (ML) Approach for AMP Prediction

#### 2.6.1 Data Collection and Labeling

The dataset for this study was constructed to perform binary classification of peptide sequences as antimicrobial peptides (AMPs) or non-antimicrobial peptides (nonAMPs). AMP sequences were obtained from the Antimicrobial Peptide Database (APD),^51^ and nonAMP sequences were collected from publicly available protein databases and curated peptide sets with no documented antimicrobial function. The final model-evaluation split used an independent held-out test set of 1,230 real peptide sequences, consisting of 609 AMPs and 621 nonAMPs, as reflected by the final confusion-matrix totals. This corresponds to an overall dataset size of 6,150 peptide sequences under the reported 80:20 split. To reduce exact or near-exact redundancy, CD-HIT^52^ was used with a 97% sequence identity threshold. The dataset was also cleaned to remove ambiguous amino acids and nonstandard characters. Each sequence was assigned a binary label, where AMPs were marked as 1 and nonAMPs as 0. We acknowledge that 97% CD-HIT filtering reduces duplicate or nearly duplicate sequences but may not fully remove homologous peptide similarity; this limitation is now discussed.

#### 2.6.2 Feature Extraction Using Composition Transition and Distribution (CTD) Scoring

To numerically encode peptide sequences, we implemented a biologically meaningful CTD scoring system.^53,54^ CTD descriptors were calculated for seven major amino acid properties, including hydrophobicity, normalized van der Waals volume, polarity, polarizability, net charge, secondary structure propensity, and solvent accessibility.^54,55^ Each property was categorized into three biochemical groups, and their distributions along peptide length were captured at five percentile positions: 0%, 25%, 50%, 75%, and 100%. This approach yielded a total of 735 numerical features per peptide sequence. Feature extraction was implemented in Python v3.12 using a custom scoring function that employed the Biopython SeqIO module to parse FASTA files. The extracted features were saved as a matrix, with each row corresponding to a peptide and each column to a CTD feature.

#### 2.6.3 Data Preprocessing and Feature Selection

Dataset preparation for model training involved standard preprocessing techniques.^56^ All numerical features were first standardized using z-score normalization, implemented via the “StandardScaler” function from the scikit-learn library. This transformation rescaled each feature to have a mean of ‘0’ and a standard deviation of 1, ensuring uniformity in feature scaling and enhancing the performance of distance-based algorithms. To mitigate multicollinearity and reduce redundancy, pairwise Pearson correlation coefficients were computed across all features. Features exhibiting a correlation coefficient greater than 0.95 were considered highly collinear, and one feature from each correlated pair was removed. This step helped preserve the interpretability and stability of the model. In addition to automated filtering, a domain-informed manual exclusion step^57^ was incorporated. Predefined features known to be irrelevant or redundant based on prior biological knowledge were excluded from the analysis to enhance model robustness. The final dataset comprised a refined subset of uncorrelated, biologically meaningful features, optimized for downstream classification tasks.

#### 2.6.4. Dataset Splitting and Class Balancing

The complete dataset was partitioned into training and test sets using an 80:20 split, with stratified sampling to preserve the AMP/non-AMP class distribution. The train-test split was performed before oversampling. SMOTE^58^ was then applied only to the training subset to reduce the small-class imbalance in the feature space. Based on the final split structure, the training set contained 4,920 peptide sequences before SMOTE. The independent test set contained 1,230 real peptide sequences and was not augmented, was not used during model fitting or hyperparameter tuning, and contained only real peptide sequences. This design was used to reduce training-set class imbalance while keeping the held-out test evaluation unbiased by synthetic samples.

#### 2.6.5 Model Training and Hyperparameter Optimization

Three supervised machine learning classifiers were developed and evaluated including Random Forest (RF),^59^ Support Vector Machine (SVM),^60^ and k Nearest Neighbors (KNN).^61^ Hyperparameter optimization was performed using RandomizedSearchCV^62^ with parameter spaces tailored to each model. For Random Forest, we optimized the number of trees, maximum depth, split criteria, and class weighting. For the SVM, we tuned the kernel type, the regularization parameter (C), the gamma value, and the class-balancing strategy. For KNN, the optimal number of neighbors, distance metric, and algorithm variant were determined. All models were validated using 5-fold cross-validation, and the best-performing configurations were retrained on the full SMOTE-augmented training dataset. Additionally, a soft voting ensemble classifier^63^ was constructed by combining the three optimized models. This ensemble used probabilistic predictions from each base classifier to produce a final prediction, increasing robustness and reducing the likelihood of errors from individual models.

For transparency, the RandomizedSearchCV procedure used five-fold cross-validation on the training data. Because no full retraining was performed during this rapid revision, we did not introduce any new optimized parameters beyond those in the original analysis. The model settings available from the original workflow are reported in the supplementary materials where available, and future releases of the analysis code should include the full search spaces, best parameters, random seeds, and software versions to improve reproducibility.

#### 2.6.6 Model Evaluation and Visualization

Each trained model was evaluated on the independent test set using accuracy, precision, recall, F1 score, confusion matrices, receiver operating characteristic (ROC) curves, and area under the ROC curve (AUC).^64^ Confusion matrices were generated to show true positives, true negatives, false positives, and false negatives. ROC curves were plotted to illustrate the trade-off between sensitivity and specificity. To improve uncertainty reporting without changing the trained models, 95% confidence intervals were calculated from the held-out confusion-matrix counts for the main performance metrics where possible. AUC confidence intervals and formal paired significance testing, such as McNemar testing for paired classification errors or DeLong/bootstrap testing for AUC differences, require access to full paired prediction probabilities and should be included in future external validation work. Feature importance analysis was performed using Gini-based importance for Random Forest and permutation-based importance for SVM and KNN.^65^

#### 2.6.7 Application to Genome Mined RiPP Like Sequences

The ensemble model was applied to RiPP-like peptides predicted through genome mining using BAGEL and antiSMASH to assess its real-world applicability. These sequences underwent CTD feature extraction and were standardized using the pre-trained StandardScaler. Feature vectors were aligned to match the model’s input format, and AMP predictions were generated, yielding both binary labels and probabilistic confidence scores. High-confidence candidates were defined as AMP-like predictions with probability_AMP ≥ 0.95. When the same sequence ID appeared multiple times, duplicate entries were collapsed to the highest AMP probability for that ID. This enabled a more stringent and consistent prioritization of candidates for subsequent experimental validation.

## 3. Results

### 3.1. ANI and Pangenomic Analysis

**Figure 2.**
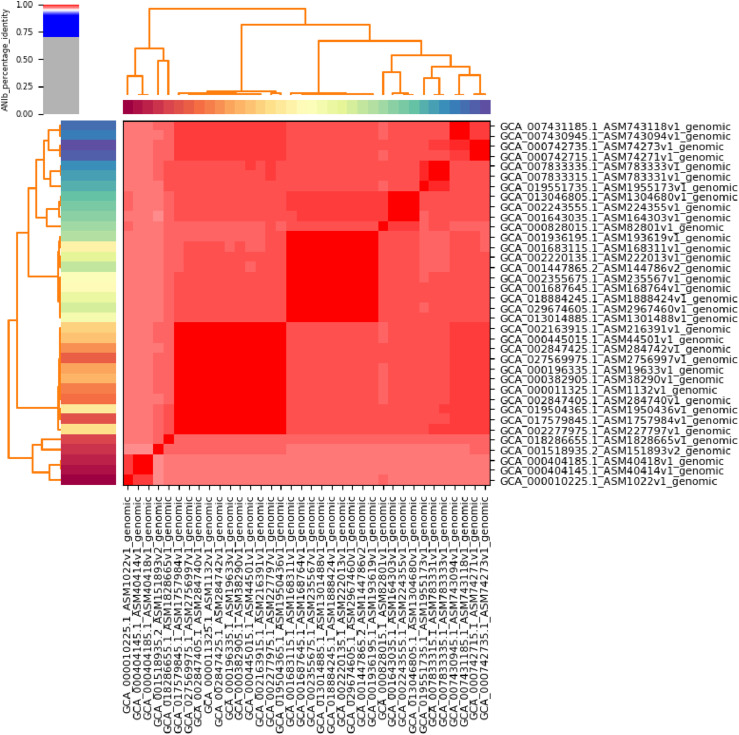
Heatmap and dendrogram of average nucleotide identity (ANI) analysis for *C. glutamicum* strains. The ANI analysis of 36 *C. glutamicum* strains shows genetic similarity, with color intensity ranging from red (indicating high ANI values) to white (indicating low ANI values). The dendrogram illustrates phylogenetic relationships, clustering strains with similar genetic characteristics

**Figure 3.**
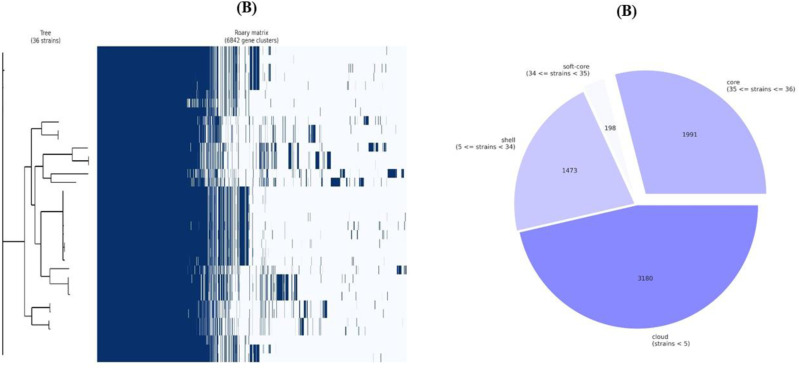
Pangenome Analysis of *C. glutamicum*. (A) Representing the presence or absence of genes across the examined genomes, alongside a corresponding phylogenetic tree, and (B) The distribution of different gene categories: core genes (1991), softcore genes (198), shell genes (1473), and cloud genes (3180)

**Figure 4.**
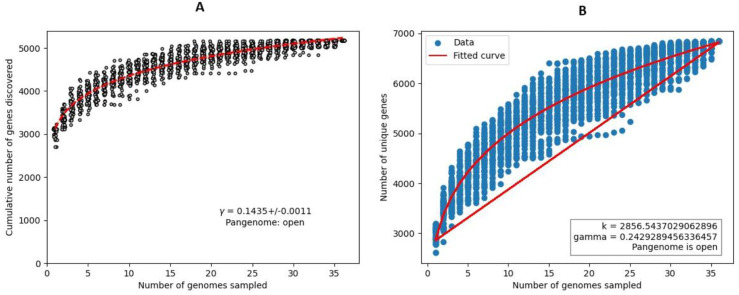
Pangenome analysis based on cumulative gene content across sampled genomes. (A) Cumulative number of genes discovered with increasing genomes sampled. The estimated γ value (0.1435 ± 0.0011) indicates an open pangenome. (B) Rarefaction curve shows the number of unique genes per additional genome. Both analyses suggest continued gene discovery and genomic diversity, supporting an open pangenome

The ANI analysis of *C. glutamicum* strains revealed a high degree of genetic relatedness among the examined genomes (Figure 2). Clustering patterns indicate similar genomic characteristics, with higher ANI values suggesting a close evolutionary relationship among most strains. This analysis effectively illustrates the genetic uniformity present within *C. glutamicum*, highlighting the potential for shared traits and functions. Hierarchical clustering reveals phylogenetic relationships, offering insights into evolutionary dynamics and genetic stability. The pangenome of *C. glutamicum* exhibits significant genetic diversity, characterized by an open pangenome structure comprising 6842 gene clusters (Figure 3A). The core genome comprises 1991 genes universally shared among strains, reflecting essential functions required for survival and metabolism. In contrast, the analysis identifies 198 softcore genes that are present in 95–99% of the strains, while 1473 shell genes occur in 15–95% of the genomes. A substantial number of cloud genes (3190) are observed, which are present in fewer than 15% of strains, indicating considerable variation in accessory functions (Figure 3B). This genetic diversity is crucial for *C. glutamicum*’s adaptability across various environments and enhances its utility in biotechnological applications, underscoring the importance of both core and accessory genes in defining the species’ functional potential. The pangenome analysis of bacteria yielded a power fit law with a coefficient of determination (R^2^) of 0.1435, indicating a moderate correlation between the number of genomes sampled and the cumulative number of genes discovered. The fitted curve, represented by the equation y = 0.1435 ± 0.0011 (Figure 4A), suggests that as the number of genomes sampled increases, the number of unique genes discovered also increases, but at a decreasing rate. Additionally, the Heaps’ law plot shows a strong positive correlation between the number of unique genes and the number of genomes sampled, with a correlation coefficient (k) of 2856.543 and a gamma value of 0.242 (Figure 4B). These results suggest that the pangenome of *C. glutamicum* is open, meaning that new genes are constantly being discovered as more genomes are sampled, and that the number of unique genes increases rapidly as more genomes are sampled.

### 3.2. BGCs in C. glutamicum Genomes

**Figure 5.**
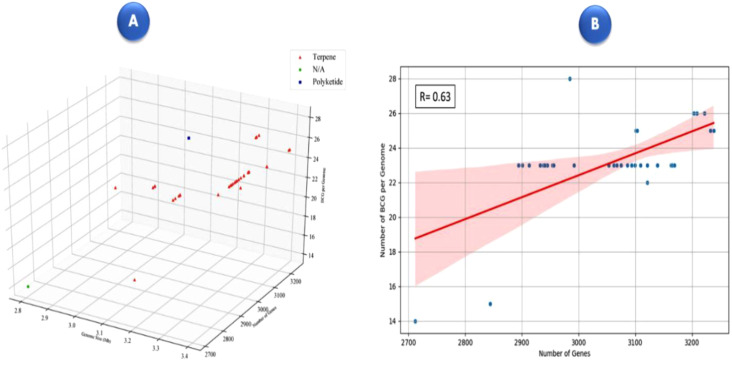
(A) 3D scatter plot representing the relationship between genome size, gene count, and the number of BGCs across 36 strains of *C. glutamicum*. The color coding reflects different biosynthetic classes. Strains with larger genomes and higher gene counts tend to have more BGCs, particularly in the Terpene class. (B) Pearson correlation plot showing a positive relationship (R = 0.63, p < 0.05) between the number of genes per genome and the number of BGCs per genome. The shaded region represents the 95% confidence interval of the linear regression model. Strains with more genes exhibit more BGCs, suggesting an association between genomic content and biosynthetic potential

**Figure 6.**
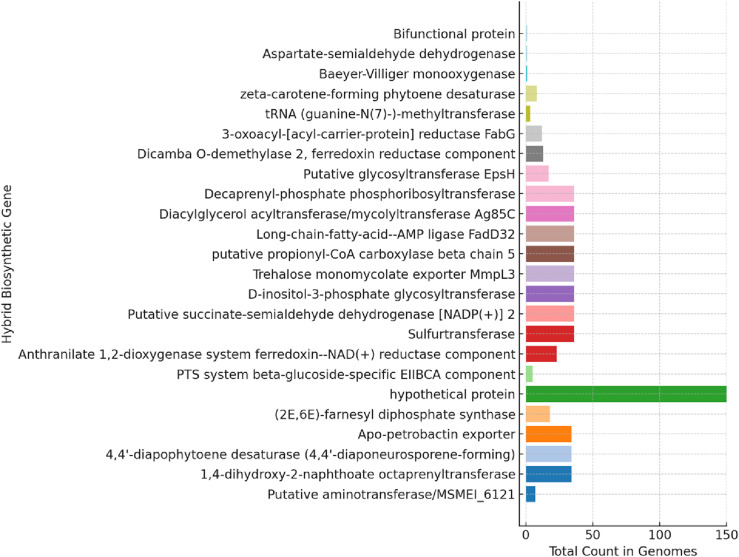
Potential hybrid BGCs are found in all the genomic strains of *C. glutamicum*

The genome sizes of the *C. glutamicum* strains ranged from 2.8 Mb to 3.4 Mb, while the number of genes varied from 2712 to 3238. Despite this genomic variation, the number of BGCs per genome remained relatively consistent, with most strains containing 23-26 BGCs. This suggests a conserved biosynthetic potential across most strains, despite differences in genome size. However, outliers such as strain B253 exhibited a higher BGC count (28 BGCs), indicating a potential for more extensive biosynthetic capabilities in these strains (Figure 5A). This result reveals that most strains harbor BGCs primarily classified as terpene-producing clusters, with a few strains containing polyketide BGCs. To quantify the relationship between genome size, gene content, and BGC abundance, we conducted a Pearson correlation analysis (Figure 5B), which showed a statistically significant positive correlation (R = 0.63, p < 0.05) between the number of genes per genome and the number of BGCs per genome. This indicates that strains with larger genomes and more genes tend to possess a greater number of BGCs, highlighting the connection between genomic expansion and biosynthetic potential. Strains such as ATCC 13032, BE, and ATCC 14067 exemplify this trend by exhibiting the highest number of BGCs (25–26 per genome). Conversely, strains with smaller genomes, such as C1 and BCA exhibit fewer BGCs (14 and 15, respectively), indicating that a reduced genome size imposes limitations on biosynthetic potential. Based on our *in silico* analysis of BGCs, the majority of BGCs were predicted to encode novel terpene compounds, including carotenoids and nocardiopsistin variants A, B, and C. Additionally, we identified hybrid BGCs that harbor genes encoding multiple types of scaffold-synthesizing enzymes, combined in various configurations. After categorizing the 11 BGCs by hybrid type, we identified 24 unique BGCs. Figure 6 shows the distribution of hybrid BGCs in different bacterial genomes. The most prevalent gene in the dataset are Sulfurtransferase, Putative succinate-semialdehyde dehydrogenase [NADP(+)] 2,D-inositol-3-phosphate glycosyltransferase, Trehalose monomycolate exporter MmpL3, putative propionyl-CoA carboxylase beta chain 5,Long-chain-fatty-acid--AMP ligase FadD32, Diacylglycerol acyltransferase/mycolyltransferase Ag85C, and Decaprenyl-phosphate phosphoribosyltransferase with 36 instances across all genomes. The second most prevalent genes are 1,4-dihydroxy-2-naphthoate octaprenyltransferase 4,4′-diapophytoene desaturase (4,4′-diaponeurosporene-forming) and Apo-petrobactin exporter, which found 34 occasions in all genome strains. The least prevalent genes in the dataset are bifunctional protein, aspartate-semialdehyde dehydrogenase, Baeyer-Villiger monooxygenase, zeta-carotene-forming phytoene desaturase, and tRNA (guanine-N(7)-)-methyltransferase that found in only B253 strain.

### 3.3. Phylogenetic Tree With BGCs Distribution

**Figure 7.**
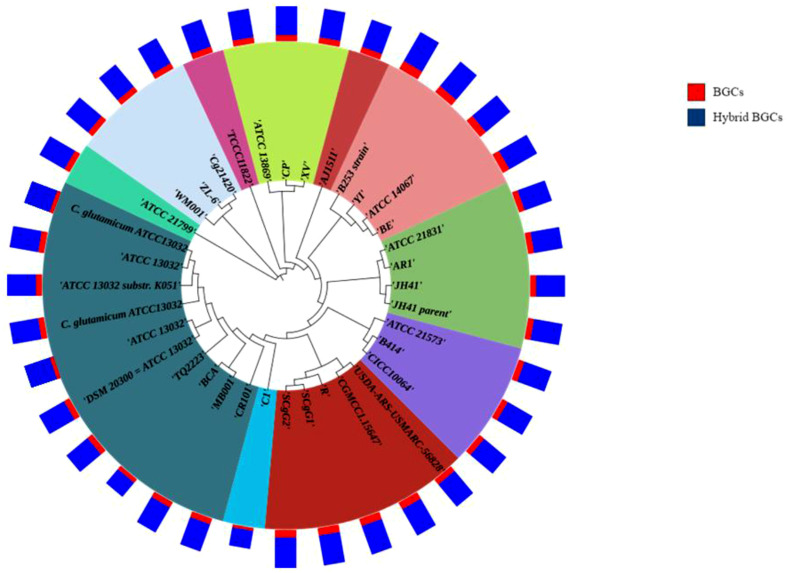
Phylogenetic tree depicting the evolutionary relationships among *C. glutamicum* strains, with branch labels corresponding to the number of biosynthetic gene clusters (BGCs) and hybrid biosynthetic gene clusters identified in each strain

The phylogenetic tree of *C. glutamicum* strains highlights evolutionary relationships and genetic divergence based on the number of BGCs and hybrid BGCs (Figure 7). Strains that cluster together in the same branches share the same number of BGCs, indicating their close evolutionary relationship and conserved biosynthetic potential. For instance, strains such as ATCC 13869, TCCC11822, and Cg21420, consistently contain 4 BGCs, underscoring a conserved metabolic capacity within this clade. Similarly, strains like ATCC 14067 and BE, share 5 BGCs. These findings suggest that strains within the same branches are more genetically stable with respect to BGC content. However, strains such as BE (21 Hybrid BGCs) and ATCC 14067 (21 Hybrid BGCs) share the same number of core BGCs but have evolved more Hybrid BGCs than strains like ATCC 13032 (19 Hybrid BGCs), which has fewer. An exception to this pattern is the B253 strain, which contains 6 BGCs and 22 hybrid BGCs, substantially more than most other strains analyzed. By comparison, strains such as C1 and BCA harbor only 2 and 3 BGCs, respectively, along with fewer hybrid BGCs (12), suggesting lower biosynthetic potential and possibly reduced adaptive capacity. Overall, the tree illustrates both the conservation of core BGCs among closely related strains and the genomic plasticity manifesting through hybrid BGC diversification.

### 3.4. RiPPs Mining

**Figure 8.**
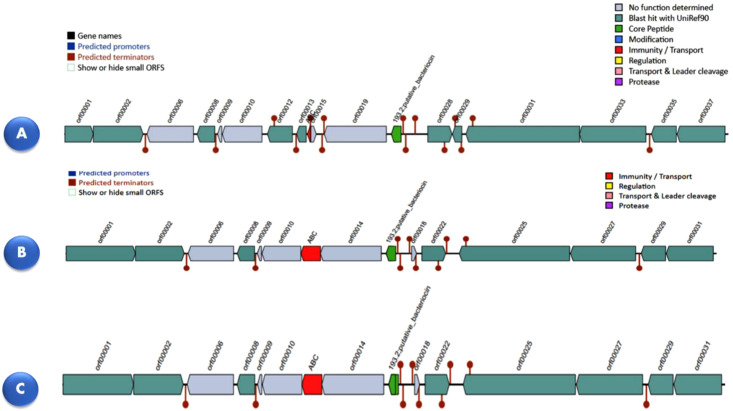
Identification of bacteriocins in three different regions from *C. glutamicum* Strains CP (A and B) and B414 (C)

We analyzed core and pangenome sequences from 36 *C. glutamicum* strains to identify RiPPs and unmodified bacteriocins. Our investigation revealed the presence of three bacteriocins (Figure 8), specifically Lactococcins, from two distinct strains CP (3,121 genes) and B414 (2938 genes). Interestingly, these two strains showed identical numbers of BGCs; however, in the phylogenetic analysis, they formed two distinct clusters, highlighting their genetic divergence. To further assess the antimicrobial potential of the identified bacteriocins, we employed three machine learning-based classifiers, namely RF, SVM, and KNN.

### 3.5. ML Model Training and Cross-Validation Performance

Each of the three classifiers, RF, SVM, and KNN, was trained to distinguish AMPs from nonAMPs using CTD-derived physicochemical features. On the independent held-out test set, Random Forest achieved an accuracy of 83.7% (95% CI: 81.6-85.7%), precision of 86.7% (95% CI: 83.6-89.3%), recall of 79.3% (95% CI: 75.9-82.3%), and F1 score of 82.8%. SVM achieved the best overall test accuracy at 84.1% (95% CI: 82.0-86.1%), with a precision of 84.9% (95% CI: 81.9-87.6%), a recall of 82.6% (95% CI: 79.4-85.4%), and an F1 score of 83.8%. KNN achieved an accuracy of 81.6% (95% CI: 79.4-83.7%), precision of 85.1% (95% CI: 81.9-87.9%), recall of 76.2% (95% CI: 72.7-79.4%), and F1 score of 80.4%. These values show that all three models performed reasonably well on the held-out test set, with SVM giving the strongest overall balance. However, because the dataset was filtered to 97% sequence identity, the performance should be interpreted as internal held-out rather than as proof of broad external generalization. Future work should repeat model training after stricter peptide clustering and validate the models on fully independent external AMP/nonAMP datasets.

### 3.6. Classifier ROC Performance

**Figure 9.**
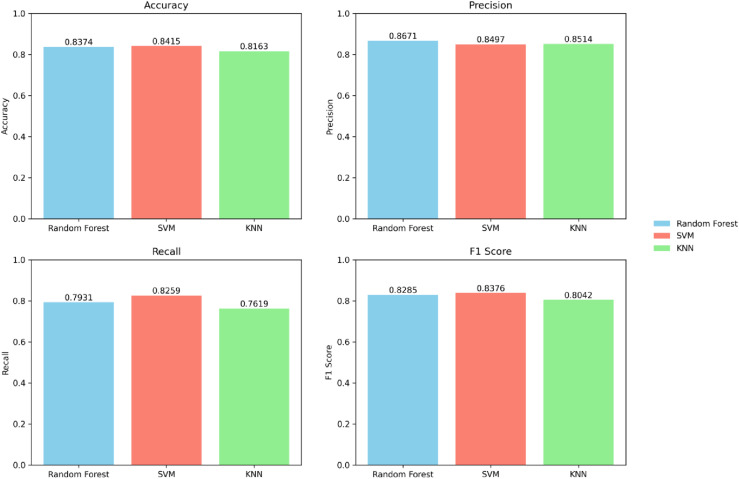
Comparative performance evaluation of three classification algorithms (RF, SVM, and KNN) for antimicrobial peptide prediction. Bar plots represent the values of four performance metrics: Accuracy, precision, recall, and F1 score. SVM achieved the highest overall performance across most metrics, particularly in accuracy (0.8415), recall (0.8259), and F1 score (0.8376). Random Forest performed best in precision (0.8671), while KNN showed comparatively lower scores across all metrics

**Figure 10.**
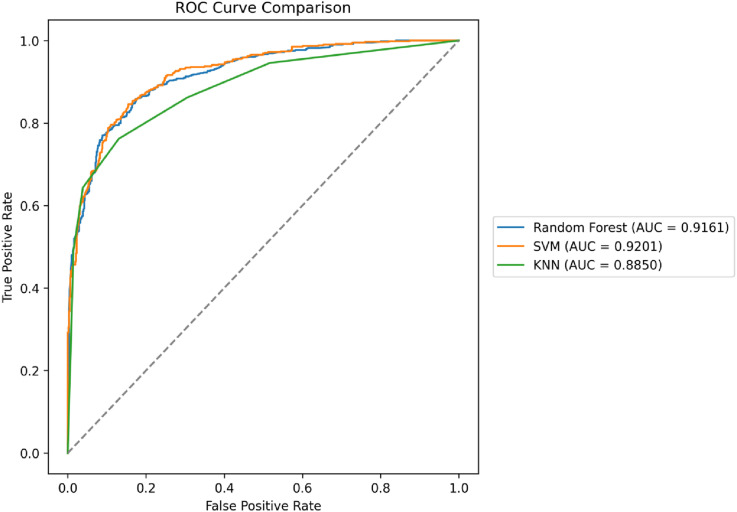
ROC curve comparison of RF, SVM, and KNN classifiers. The plot illustrates the trade-off between true positive rate (sensitivity) and false positive rate for each model. The AUC values are indicated in the legend: SVM (0.9201), Random Forest (0.9161), and KNN (0.8850). AUC values closer to 1.0 indicate better overall classification performance. SVM showed the highest AUC, demonstrating the most effective discrimination between AMPs and nonAMPs among the tested models

The ROC curves illustrate the classification performance of RF, SVM, and KNN in distinguishing AMPs from non-AMPs. SVM achieved the highest AUC (0.9201), indicating superior classification performance across all decision thresholds (Figure 9). RF also demonstrated strong discriminative ability with an AUC of 0.9161 (Figure 10). KNN, however, yielded a comparatively lower AUC (0.8850) (Figure 10), suggesting it was less effective in distinguishing between classes. These findings highlight that while all three models performed reasonably well, SVM offered the best overall accuracy in terms of sensitivity-specificity balance. In addition, the small performance gap between SVM and RF suggests both models are suitable for AMP prediction tasks, with SVM having a slight edge in classification robustness.

### 3.7. Model Interpretability and Feature Importance

**Figure 11.**
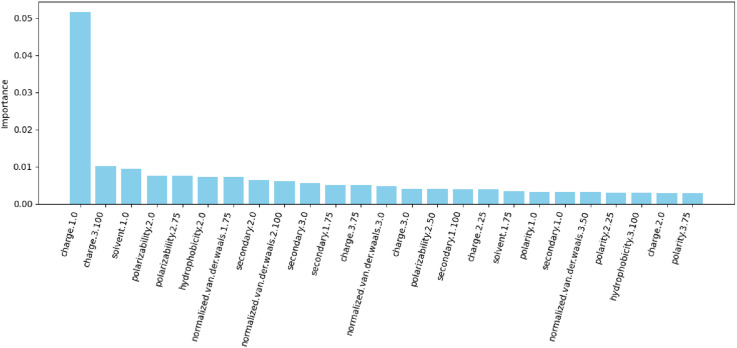
Feature importance ranking for SVM classifier in AMP prediction

Figure 11 illustrates the 25 most influential features contributing to the SVM model for distinguishing AMPs from nonAMPs. The analysis shows that electrostatic charge-related descriptors were among the strongest contributors to classification. This is biologically reasonable because many AMPs are cationic and interact with negatively charged bacterial membranes. In addition, hydrophobicity, polarizability, solvent accessibility, normalized van der Waals volume, and secondary-structure descriptors suggest that amphipathic organization and residue distribution may contribute to AMP-like behavior. These findings provide a useful biological interpretation of the model, but they should be treated as mechanistic hypotheses only. The present analysis does not prove peptide folding, membrane disruption, bond cleavage, metabolite production, or antimicrobial activity; these points require experimental and structural validation.

### 3.8. Assessment of Model Performance

**Figure 12.**
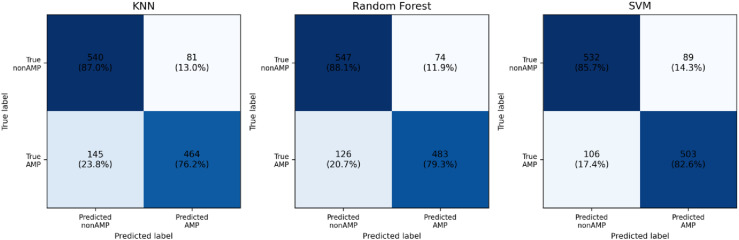
Confusion matrices for KNN (A), Random Forest (B), and SVM (C) classifiers. Each cell shows the absolute count and the row-normalized percentage within the true class. The matrices show classification outcomes for AMP prediction; SVM achieved the highest true positive count, while KNN had the highest false negative rate

**Figure 13.**
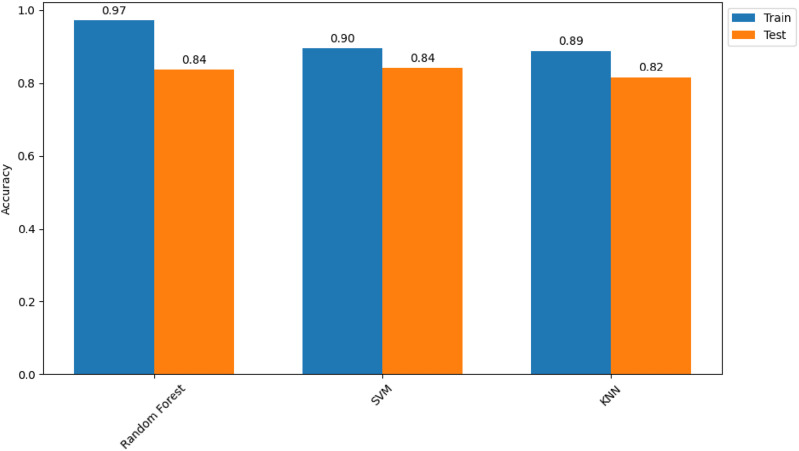
Comparison of training and test accuracy across classifiers. SVM showed the most consistent performance between training and test sets, while Random Forest exhibited signs of overfitting and KNN showed the lowest accuracy overall

The confusion matrices for the three classifiers KNN (Figure 12A), Random Forest (Figure 12B), and SVM (Figure 12C) provide detailed insights into prediction performance by illustrating the true positives, true negatives, false positives, and false negatives. While Random Forest correctly classified 547 negative and 483 positive instances, it misclassified 74 negatives and 126 positives. In contrast, SVM slightly improved positive class detection with 503 true positives, though at the cost of increased false positives (89). KNN, however, showed the lowest performance, with the highest number of false negatives (145), indicating its lower sensitivity to detecting AMPs. Overall, SVM demonstrates the most balanced confusion matrix with the highest true positive rate, while Random Forest maintains slightly better specificity. KNN, despite reasonable accuracy, underperforms in recall, particularly for AMP detection. As shown in Figure 13, the Support Vector Machine (SVM) classifier exhibited the most consistent performance, achieving 0.90 training accuracy and 0.84 test accuracy, indicating strong generalization with minimal overfitting. In contrast, Random Forest achieved the highest training accuracy **(**0.97**)** but experienced a noticeable drop on the test set **(**0.84**),** suggesting potential overfitting. K-Nearest Neighbors (KNN) recorded the lowest performance overall, with 0.89 training and 0.82 test accuracy, reflecting reduced generalization capacity.

### 3.9. Prediction of Novel AMP-like Candidates

The trained ensemble classifier was applied to RiPP-like peptides identified from BAGEL5 and antiSMASH genome-mining outputs. All peptide sequences were processed using the same CTD-based feature-extraction pipeline and standardized using the pre-trained scaler prior to prediction. The model-generated AMP probability scores were subsequently used to prioritize candidate peptides for further investigation. A total of 21 prediction entries exceeded the stringent high-confidence threshold (AMP probability ≥ 0.95). As several sequence identifiers were represented multiple times, duplicate entries were collapsed by retaining the sequence with the highest predicted AMP probability for each identifier. This filtering strategy identified 18 unique AMP-like candidates as high-confidence computational leads. However, these candidates represent in silico predictions only and should not be considered experimentally validated antimicrobial peptides. Therefore, the predicted functional relevance of these candidates remains preliminary, and their antimicrobial activity, cytotoxicity, stability, spectrum of activity, and mechanisms of action require comprehensive experimental validation in future studies.

**Figure 14.**
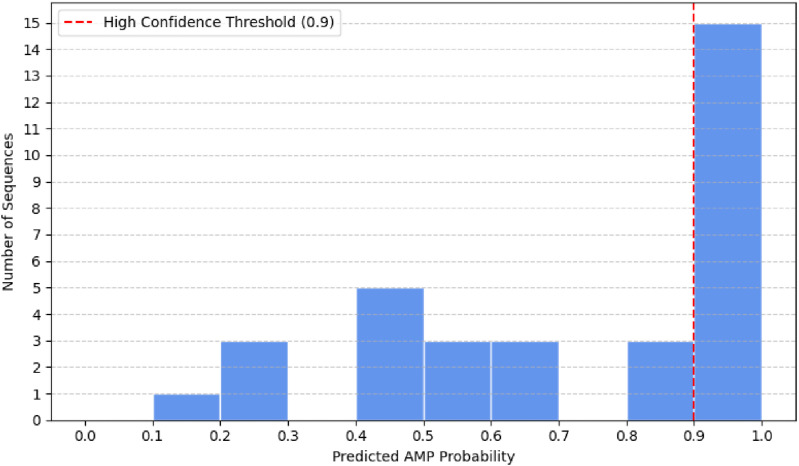
Predicted AMP probability distribution for novel RiPP-like peptides. The figure shows the distribution of predicted AMP probabilities after duplicate sequence IDs were collapsed by keeping the highest probability value. The red dashed line indicates the high-confidence threshold (probability_AMP = 0.95).Figure 14 Candidates at or above this threshold were considered high-confidence AMP-like candidates for experimental prioritization

## 4. Discussion

This study presents the first integrative analysis combining comparative pangenomics, biosynthetic gene cluster (BGC) mining, and machine learning (ML)–based AMP prediction in *C. glutamicum*. While *C. glutamicum* has long been established as a safe and efficient industrial workhorse for amino acid production,^66^ its potential for natural product biosynthesis has been comparatively underexplored.^67^ By analyzing 36 complete genomes, we demonstrate that *C. glutamicum* possesses an open pangenome characterized by a robust core genome and a highly variable accessory genome. This genetic diversity underpins its metabolic adaptability and highlights an untapped reservoir of secondary metabolite biosynthetic capacity. The finding that genome expansion correlates positively with BGC abundance supports prior studies in other bacteria, where genomic plasticity enhances natural product diversity.^68,69^ Such adaptability is crucial in ecological and industrial contexts, allowing this species to thrive in diverse environments and offering promising avenues for biotechnological exploitation.

A major insight from this study is the consistent presence of terpene-producing clusters across strains, alongside the discovery of polyketide and hybrid BGCs. Terpenes are widely recognized for their applications in pharmaceuticals, nutraceuticals, and agriculture,^70,71^ and their predominance suggests *C. glutamicum* could serve as a platform for industrial-scale terpene production, complementing recent efforts in metabolic engineering.^71^ More strikingly, the identification of polyketide synthase (PKS)–associated BGCs is significant, as polyketides are among the most pharmacologically valuable secondary metabolites, including antibiotics, antifungals, and immunosuppressants.^72^ Polyketides have not previously been reported in *C. glutamicum*, making this observation a novel contribution to its biosynthetic repertoire. Furthermore, the detection of hybrid BGCs that combine multiple biosynthetic logics underscores the metabolic versatility of C. glutamicum. Hybrid clusters are known to drive structural diversification of metabolites in *Streptomyces* and *Burkholderia*,^73,74^ and their presence here suggests similar potential for generating chemically novel scaffolds. Outlier strains such as B253, which contained an unusually high number of hybrid BGCs, may represent evolutionary lineages undergoing extensive genetic rearrangements, offering particularly rich opportunities for novel metabolite discovery.

The mining of RiPPs further illustrates *C. glutamicum*’s hidden biosynthetic capacity. RiPPs, including bacteriocins such as Lactococcins identified in this study, represent a promising class of antimicrobial molecules with significant therapeutic potential.^46,75^ The detection of three Lactococcin variants in two phylogenetically distinct strains (CP and B414) highlights both evolutionary divergence and metabolic flexibility within this species. Interestingly, although these strains shared identical BGC counts, they occupied separate clades in the phylogenetic analysis, suggesting regulatory or evolutionary differences that may influence metabolite expression. Previous work has demonstrated the successful recombinant production of bacteriocins such as garvicin Q and pediocin PA-1 in *C. glutamicum*,^76^ supporting the feasibility of leveraging this organism for bacteriocin discovery and production. Our results extend these capabilities by identifying endogenous RiPP-like clusters with strong in silico AMP potential, positioning *C. glutamicum* as a valuable reservoir for antimicrobial peptide discovery.^77^

A critical advancement of this study lies in the integration of machine learning into the genome-mining pipeline. Conventional tools such as antiSMASH and BAGEL5 are indispensable for detecting known BGC and RiPP signatures, but they rely largely on homology, curated rules, and pHMM-based recognition.^9,42^ Such approaches may miss divergent AMP-like peptides that have weak similarity to known families. By contrast, CTD-based ML uses sequence-derived physicochemical features, including charge, hydrophobicity, polarity, polarizability, solvent accessibility, van der Waals volume, and secondary-structure tendency. This does not replace genome mining, but it adds a complementary prioritization layer for identifying peptide candidates with AMP-like biochemical properties. In the current analysis, SVM, RF, and KNN all achieved held-out accuracies above 81%, with SVM showing the strongest balance across metrics. These results are broadly consistent with previous AMP prediction studies, although direct comparison is limited because different studies use different datasets, redundancy thresholds, feature encodings, and validation designs. Therefore, the ML results should be viewed as a candidate-prioritization tool rather than a final biological validation step.

The significance of this work is twofold. First, it positions *C. glutamicum* a GRAS organism with well-established industrial tractability as an untapped resource for natural product biosynthesis. This dual utility strengthens its potential as both a chassis for synthetic biology and a source of endogenous bioactive compounds. Second, by integrating ML into pangenome mining, this study provides a scalable framework that can be extended to other microbial genera, particularly those with underexplored genomic diversity. In the context of rising antimicrobial resistance, this approach offers a computationally guided strategy for accelerating the identification of novel antimicrobial agents.^76^

Several limitations should be considered. First, the predicted AMP candidates are computationally prioritized leads and should not be interpreted as experimentally confirmed antimicrobial peptides. The present study did not include peptide synthesis, minimum inhibitory concentration assays, cytotoxicity or hemolysis testing, LC-MS/MS metabolomics, structural modeling, or membrane-interaction experiments. These analyses are required to confirm biological activity, toxicity, structural stability, metabolite products, and the mechanism of action. Second, although CD-HIT filtering at 97% identity removed exact or near-exact duplicates, this threshold may not fully eliminate homologous peptide similarity between the training and test sets. Such redundancy can inflate apparent model performance. Future work should repeat model development after stricter sequence clustering, such as 70% or 40% identity, and should evaluate performance on independent external AMP/nonAMP datasets. Third, the genome analysis was limited to complete publicly available genomes, which improves BGC reliability but may not capture the full environmental or clinical diversity of *C. glutamicum*. Broader genome sampling, transcriptomics, metabolomics, structural modeling, and wet-lab validation will be needed to fully assess the biosynthetic and antimicrobial potential of the prioritized candidates.

## 5. Conclusion

This work expands the known biosynthetic landscape of *C. glutamicum* by combining pangenome analysis, genome mining, and ML-based prediction of AMP-like peptides. Although *C. glutamicum* is mainly known for industrial amino acid production, the analyzed genomes contained diverse biosynthetic gene clusters, including hybrid and RiPP-like clusters with underexplored potential. The detection of polyketide-associated clusters is especially notable, as these clusters have not been widely recognized in this species. By training ML classifiers on CTD physicochemical sequence features and using a stricter high-confidence probability threshold of 0.95, this study prioritized 18 unique AMP-like candidates for future validation. The results support *C. glutamicum* as a promising source of bioactive metabolite candidates and show that ML can help narrow the search space for antimicrobial discovery. At the same time, the findings remain computational and require stricter external validation, structural analysis, and experimental testing before biological or therapeutic claims can be made.

## Supplemental Material

Supplemental Material - Hidden in the Pangenome? Machine Learning-Driven Discovery of Antimicrobial Potential in *Corynebacterium glutamicum* Supplemental Material for Hidden in the Pangenome? Machine Learning-Driven Discovery of Antimicrobial Potential in *Corynebacterium glutamicum* by Sk Injamamul Islam, Khandker Shahed, Mst. Mahmuda Parvin, Saloa Sanjida in Bioinformatics and Biology Insights.

Supplemental Material - Hidden in the Pangenome? Machine Learning-Driven Discovery of Antimicrobial Potential in *Corynebacterium glutamicum* Supplemental Material for Hidden in the Pangenome? Machine Learning-Driven Discovery of Antimicrobial Potential in *Corynebacterium glutamicum* by Sk Injamamul Islam, Khandker Shahed, Mst. Mahmuda Parvin, Saloa Sanjida in Bioinformatics and Biology Insights.

## Data Availability

Genome accession numbers, BGC summary data, and peptide-prediction outputs used in this study are provided in the manuscript and supplementary materials. Additional processed files or scripts are available from the corresponding author upon reasonable request.*

## Appendix

### Abbreviations

AMP: Antimicrobial peptide
ANI: Average nucleotide identity
APD: Antimicrobial Peptide Database
AUC: Area under the receiver operating characteristic curve
BAGEL: Bacteriocin genome-mining tool
BGC: Biosynthetic gene cluster
CD-HIT: Cluster Database at High Identity with Tolerance
CTD: Composition, transition, and distribution
FN: False negative
FP: False positive
KNN: K-nearest neighbors
ML: Machine learning
nonAMP: Non-antimicrobial peptide
pHMM: Profile hidden Markov model
PKS: Polyketide synthase
RF: Random Forest
RiPP: Ribosomally synthesized and post-translationally modified peptide
ROC: Receiver operating characteristic
SMOTE: Synthetic Minority Oversampling Technique
SVM: Support vector machine
TN: True negative
TP: True positive.

## References

[1] Rutledge PJ Challis GL . Discovery of microbial natural products by activation of silent biosynthetic gene clusters. Nature Reviews Microbiology. 2015;13(8):509-523. 26119570 10.1038/nrmicro3496

[2] Newman DJ Cragg GM . Natural Products as Sources of New Drugs over the Nearly Four Decades from 01/1981 to 09/2019. Journal of Natural Products. 2020;83(3):770-803. 32162523 10.1021/acs.jnatprod.9b01285

[3] Kalkreuter E Pan G Cepeda AJ Shen B . Targeting bacterial genomes for natural product discovery. Trends in Pharmacological Sciences. 2020;41(1):13-26. 31822352 10.1016/j.tips.2019.11.002 PMC6938545

[4] Chevrette MG Carlson CM Ortega HE , et al. The antimicrobial potential of Streptomyces from insect microbiomes. Nature communications. 2019;10(1):516. 10.1038/s41467-019-08438-0 PMC6355912 30705269

[5] Baltz RH . Natural product drug discovery in the genomic era: realities, conjectures, misconceptions, and opportunities. Journal of Industrial Microbiology and Biotechnology. 2019;46(3-4):281-299. 30484124 10.1007/s10295-018-2115-4

[6] Genilloud O . Mining actinomycetes for novel antibiotics in the omics era: are we ready to exploit this new paradigm? Antibiotics. 2018;7(4):85. 30257490 10.3390/antibiotics7040085 PMC6316141

[7] Xu F Wu Y Zhang C , et al. A genetics-free method for high-throughput discovery of cryptic microbial metabolites. Nature chemical biology. 2019;15(2):161-168. 30617293 10.1038/s41589-018-0193-2 PMC6339573

[8] Baltz RH . Gifted microbes for genome mining and natural product discovery. Journal of Industrial Microbiology and Biotechnology. 2017;44(4-5):573-588. 27520548 10.1007/s10295-016-1815-x

[9] Blin K Shaw S Kloosterman AM , et al. antiSMASH 6.0: improving cluster detection and comparison capabilities. Nucleic acids research. 2021;49(W1):W29-W35. 33978755 10.1093/nar/gkab335 PMC8262755

[10] Yang J Yang S . Comparative analysis of Corynebacterium glutamicum genomes: a new perspective for the industrial production of amino acids. BMC Genomics. 2017;18(1):940. 28198668 10.1186/s12864-016-3255-4 PMC5310272

[11] Kalinowski J Bathe B Bartels D , et al. The complete Corynebacterium glutamicum ATCC 13032 genome sequence and its impact on the production of l-aspartate-derived amino acids and vitamins. Journal of Biotechnology. 2003;104(1):5-25. 12948626 10.1016/s0168-1656(03)00154-8

[12] Feierabend M Renz A Zelle E Nöh K Wiechert W Dräger A . High-Quality Genome-Scale Reconstruction of Corynebacterium glutamicum ATCC 13032. Frontiers in Microbiology. 2021;12:750206. 34867870 10.3389/fmicb.2021.750206 PMC8634658

[13] Lin K Han S Zheng S . Application of Corynebacterium glutamicum engineering display system in three generations of biorefinery. Microbial Cell Factories. 2022;21(1):14. 35090458 10.1186/s12934-022-01741-4 PMC8796525

[14] Wittmann C Krull R . Biosystems engineering I: Creating superior biocatalysts, 120. Springer Science & Business Media; 2010.

[15] Vertès AA Inui M Yukawa H . The biotechnological potential of Corynebacterium glutamicum, from Umami to Chemurgy. Corynebacterium glutamicum: Biology and Biotechnology. 2013:1-49.

[16] Arnone JT . Genomic considerations for the modification of Saccharomyces cerevisiae for biofuel and metabolite biosynthesis. Microorganisms. 2020;8(3):321. 32110897 10.3390/microorganisms8030321 PMC7143498

[17] Liu X Yang Y Zhang W , et al. Expression of recombinant protein using Corynebacterium glutamicum: progress, challenges and applications. Critical reviews in biotechnology. 2016;36(4):652-664. 25714007 10.3109/07388551.2015.1004519

[18] Vertes AA . Protein secretion systems of Corynebacterium glutamicum. In: Corynebacterium glutamicum: Biology and biotechnology. Springer; 2012:351-389.

[19] Baritugo KAG Kim HT David YC , et al. Recent advances in metabolic engineering of Corynebacterium glutamicum as a potential platform microorganism for biorefinery. Biofuels, Bioproducts and Biorefining. 2018;12(5):899-925.

[20] Kogure T Inui M . Recent advances in metabolic engineering of Corynebacterium glutamicum for bioproduction of value-added aromatic chemicals and natural products. Applied microbiology and biotechnology. 2018;102:8685-8705. 30109397 10.1007/s00253-018-9289-6

[21] Conrady M Lemoine A Limberg MH Oldiges M Neubauer P Junne S . Carboxylic acid consumption and production by Corynebacterium glutamicum. Biotechnology progress. 2019;35(3):e2804. 30851150 10.1002/btpr.2804

[22] Chen C Pan J Yang X , et al. Global transcriptomic analysis of the response of Corynebacterium glutamicum to ferulic acid. Archives of microbiology. 2017;199:325-334. 27766354 10.1007/s00203-016-1306-5

[23] Choi JW Jeon EJ Jeong KJ . Recent advances in engineering Corynebacterium glutamicum for utilization of hemicellulosic biomass. Current opinion in biotechnology. 2019;57:17-24. 30537644 10.1016/j.copbio.2018.11.004

[24] Kitade Y Hiraga K Inui M . Aromatic compound catabolism in Corynebacterium glutamicum. Corynebacterium glutamicum: Biology and Biotechnology. 2020:323-337.

[25] Yan J Cai J Zhang B Wang Y Wong DF Siu SWI . Recent progress in the discovery and design of antimicrobial peptides using traditional machine learning and deep learning. Antibiotics. 2022;11(10):1451. 36290108 10.3390/antibiotics11101451 PMC9598685

[26] Veltri D Kamath U Shehu A . Deep learning improves antimicrobial peptide recognition. Bioinformatics. 2018;34(16):2740-2747. 29590297 10.1093/bioinformatics/bty179 PMC6084614

[27] Fjell CD Jenssen H Hilpert K , et al. Identification of novel antibacterial peptides by chemoinformatics and machine learning. Journal of medicinal chemistry. 2009;52(7):2006-2015. 19296598 10.1021/jm8015365

[28] Shahed K Islam SI Sangsawad P , et al. Benchmarking pangenome dynamics and horizontal gene transfer in Mycobacterium marinum evolution. Frontiers in Microbiology. 2025;16:16-2025. 10.3389/fmicb.2025.1537826 PMC12209367 40600140

[29] Wu H Wang D Gao F . Toward a high-quality pan-genome landscape of Bacillus subtilis by removal of confounding strains. Briefings in Bioinformatics. 2021;22(2):1951-1971. 32065216 10.1093/bib/bbaa013

[30] Wu H Yang ZK Yang T , et al. An effective preprocessing method for high-quality pan-genome analysis of Bacillus subtilis and Escherichia coli. Essential Genes and Genomes: Methods and Protocols. 2022:371-390. 10.1007/978-1-0716-1720-5_21 34709628

[31] Shahed K Chakma A Manjur OHB Islam SI . Multiscale comparative pathogenomic analysis of Vibrio anguillarum linking serotype diversity, genomic plasticity and pathogenicity. Journal of Genetic Engineering and Biotechnology. 2025;23(3):100522. 40854641 10.1016/j.jgeb.2025.100522 PMC12210308

[32] Kurtz S Phillippy A Delcher AL , et al. Versatile and open software for comparing large genomes. Genome biology. 2004;5:1-9. 10.1186/gb-2004-5-2-r12 PMC395750 14759262

[33] Camacho C Coulouris G Avagyan V , et al. BLAST+: architecture and applications. BMC bioinformatics. 2009;10:1-9. 20003500 10.1186/1471-2105-10-421 PMC2803857

[34] Pritchard L Glover RH Humphris S Elphinstone JG Toth IK . Genomics and Taxonomy in Diagnostics for Food Security: soft-rotting enterobacterial plant pathogens. Anal. Methods. 2015;8:12-24.

[35] Abdella B Abozahra NA Shokrak NM Mohamed RA El-Helow ER . Whole spectrum of Aeromonas hydrophila virulence determinants and the identification of novel SNPs using comparative pathogenomics. Scientific Reports. 2023;13(1):7712. 37173388 10.1038/s41598-023-34887-1 PMC10182093

[36] Podrzaj L Burtscher J Domig KJ . Comparative genomics provides insights into genetic diversity of Clostridium tyrobutyricum and potential implications for late blowing defects in cheese. Frontiers in Microbiology. 2022;13:889551. 35722315 10.3389/fmicb.2022.889551 PMC9201417

[37] Page AJ Cummins CA Hunt M , et al. Roary: rapid large-scale prokaryote pan genome analysis. Bioinformatics. 2015;31(22):3691-3693. 26198102 10.1093/bioinformatics/btv421 PMC4817141

[38] Rajput A Chauhan SM Mohite OS , et al. Pangenome analysis reveals the genetic basis for taxonomic classification of the Lactobacillaceae family. Food Microbiology. 2023;115:104334. 37567624 10.1016/j.fm.2023.104334

[39] Tettelin H Masignani V Cieslewicz MJ , et al. Genome analysis of multiple pathogenic isolates of Streptococcus agalactiae: Implications for the microbial “pan-genome”. Proceedings of the National Academy of Sciences. 2005;102(39):13950-13955. 10.1073/pnas.0506758102 PMC1216834 16172379

[40] Horsfield ST Tonkin-Hill G Croucher NJ Lees JA . Accurate and fast graph-based pangenome annotation and clustering with ggCaller. 2023. bioRxiv:2023. 01.24.524926. 10.1101/gr.277733.123 PMC10620059 37620118

[41] Soares SC Silva A Trost E , et al. The pan-genome of the animal pathogen Corynebacterium pseudotuberculosis reveals differences in genome plasticity between the biovar ovis and equi strains. PLoS One. 2013;8(1):e53818. 23342011 10.1371/journal.pone.0053818 PMC3544762

[42] Blin K Shaw S Augustijn HE , et al. antiSMASH 7.0: new and improved predictions for detection, regulation, chemical structures and visualisation. Nucleic Acids Research. 2023;51(W1):W46-W50. 37140036 10.1093/nar/gkad344 PMC10320115

[43] Eddy SR . Profile hidden Markov models. Bioinformatics (Oxford, England). 1998;14(9):755-763. 9918945 10.1093/bioinformatics/14.9.755

[44] Medema MH Blin K Cimermancic P , et al. antiSMASH: rapid identification, annotation and analysis of secondary metabolite biosynthesis gene clusters in bacterial and fungal genome sequences. Nucleic acids research. 2011;39(suppl_2):W339-W346. 21672958 10.1093/nar/gkr466 PMC3125804

[45] Cane DE Ikeda H . Exploration and mining of the bacterial terpenome. Accounts of chemical research. 2012;45(3):463-472. 22039990 10.1021/ar200198d PMC3288161

[46] Arnison PG Bibb MJ Bierbaum G , et al. Ribosomally synthesized and post-translationally modified peptide natural products: overview and recommendations for a universal nomenclature. Nat Prod Rep. 2013;30(1):108-160. 23165928 10.1039/c2np20085f PMC3954855

[47] Dunbar KL Melby JO Mitchell DA . YcaO domains use ATP to activate amide backbones during peptide cyclodehydrations. Nature chemical biology. 2012;8(6):569-575. 22522320 10.1038/nchembio.944 PMC3428213

[48] Stamatakis A . RAxML version 8: a tool for phylogenetic analysis and post-analysis of large phylogenies. Bioinformatics. 2014;30(9):1312-1313. 24451623 10.1093/bioinformatics/btu033 PMC3998144

[49] Letunic I Bork P . Interactive Tree Of Life (iTOL) v5: an online tool for phylogenetic tree display and annotation. Nucleic acids research. 2021;49(W1):W293-W296. 33885785 10.1093/nar/gkab301 PMC8265157

[50] van Heel AJ de Jong A Song C Viel JH Kok J Kuipers OP . BAGEL4: a user-friendly web server to thoroughly mine RiPPs and bacteriocins. Nucleic acids research. 2018;46(W1):W278-W281. 29788290 10.1093/nar/gky383 PMC6030817

[51] Wang Z Wang G . APD: the antimicrobial peptide database. Nucleic acids research. 2004;32(suppl_1):D590-D592. 14681488 10.1093/nar/gkh025 PMC308759

[52] Fu L Niu B Zhu Z Wu S Li W . CD-HIT: accelerated for clustering the next-generation sequencing data. Bioinformatics. 2012;28(23):3150-3152. 23060610 10.1093/bioinformatics/bts565 PMC3516142

[53] Govindan G Nair AS . Composition, Transition and Distribution (CTD)—a dynamic feature for predictions based on hierarchical structure of cellular sorting. In: 2011 annual IEEE India conference. Ieee; 2011.

[54] Dubchak I Muchnik I Holbrook SR Kim SH . Prediction of protein folding class using global description of amino acid sequence. Proceedings of the National Academy of Sciences. 1995;92(19):8700-8704. 10.1073/pnas.92.19.8700 PMC41034 7568000

[55] Tan J-X Li SH Zhang ZM , et al. Identification of hormone binding proteins based on machine learning methods. Math. Biosci. Eng. 2019;16(4):2466-2480. 31137222 10.3934/mbe.2019123

[56] Kang M Tian J . Machine learning: Data pre‐processing. Prognostics and health management of electronics: fundamentals, machine learning, and the internet of things. 2018:111-130.

[57] Bada AB Garko A.B. Gabi D Argungu MS . The Role of Domain Knowledge in Feature Selection for Machine Learning. Journal of Institutional Research, Big Data Analytics and Innovation. 2025;1(3):180-187.

[58] Larsen BS . Synthetic minority over-sampling technique (SMOTE). GitHub. 2022. https://github. com/dkbsl/matlab_smote/releases/tag/1.0.

[59] Belgiu M Drăguţ L . Random forest in remote sensing: A review of applications and future directions. ISPRS journal of photogrammetry and remote sensing. 2016;114:24-31.

[60] Pisner DA Schnyer DM . Support vector machine. In: Machine learning. Elsevier; 2020:101-121.

[61] Steinbach M Tan P-N . kNN: k-nearest neighbors. In: The top ten algorithms in data mining. Chapman and Hall/CRC; 2009:165-176.

[62] Aljuboori A Abdulrazzq M . Enhancing Accuracy in Predicting Continuous Values through Regression. Iraqi Journal for Computer Science and Mathematics. 2024;5(4):25.

[63] Kumari S Kumar D Mittal M . An ensemble approach for classification and prediction of diabetes mellitus using soft voting classifier. International Journal of Cognitive Computing in Engineering. 2021;2:40-46.

[64] Gönen M . Receiver operating characteristic (ROC) curves. SAS Users Group International (SUGI). 2006;31:210-231.

[65] Menze BH Kelm BM Masuch R , et al. A comparison of random forest and its Gini importance with standard chemometric methods for the feature selection and classification of spectral data. BMC Bioinformatics. 2009;10(1):213. 19591666 10.1186/1471-2105-10-213 PMC2724423

[66] Kinoshita S Udaka S Shimono M . Amino acid fermentation. I. Production of L-glutamic acid by various microorganisms. J Gen Appl Microbiol. 1957;3(1):193-205. 15965888

[67] Ikeda M Nakagawa S . The Corynebacterium glutamicum genome: features and impacts on biotechnological processes. Applied microbiology and biotechnology. 2003;62(2):99-109. 12743753 10.1007/s00253-003-1328-1

[68] Mohite OS Lloyd CJ Monk JM Weber T Palsson BO . Pangenome analysis of Enterobacteria reveals richness of secondary metabolite gene clusters and their associated gene sets. Synthetic and Systems Biotechnology. 2022;7(3):900-910. 35647330 10.1016/j.synbio.2022.04.011 PMC9125672

[69] Pizarro D Divakar PK Grewe F Crespo A Dal Grande F Lumbsch HT . Genome-wide analysis of biosynthetic gene cluster reveals correlated gene loss with absence of usnic acid in lichen-forming fungi. Genome Biology and Evolution. 2020;12(10):1858-1868. 33151307 10.1093/gbe/evaa189 PMC7643366

[70] Fan M Yuan S Li L , et al. Application of terpenoid compounds in food and pharmaceutical products. Fermentation. 2023;9(2):119.

[71] Luckie BA Kashyap M Pearson AN , et al. Development of Corynebacterium glutamicum as a monoterpene production platform. Metabolic Engineering. 2024;81:110-122. 38056688 10.1016/j.ymben.2023.11.009

[72] Jenke-Kodama H Sandmann A Müller R Dittmann E . Evolutionary Implications of Bacterial Polyketide Synthases. Molecular Biology and Evolution. 2005;22(10):2027-2039. 15958783 10.1093/molbev/msi193

[73] Alam K Islam MM Gong K , et al. In silico genome mining of potential novel biosynthetic gene clusters for drug discovery from Burkholderia bacteria. Comput Biol Med. 2022;140:105046. 34864585 10.1016/j.compbiomed.2021.105046

[74] Caicedo-Montoya C Manzo-Ruiz M Ríos-Estepa R . Pan-Genome of the Genus Streptomyces and Prioritization of Biosynthetic Gene Clusters With Potential to Produce Antibiotic Compounds. Front Microbiol. 2021;12:677558. 34659136 10.3389/fmicb.2021.677558 PMC8510958

[75] Xuan J Feng W Wang J , et al. Antimicrobial peptides for combating drug-resistant bacterial infections. Drug Resist Updat. 2023;68:100954. 36905712 10.1016/j.drup.2023.100954

[76] Desiderato CK Müller C Schretzmeier A , et al. Optimized recombinant production of the bacteriocin garvicin Q by Corynebacterium glutamicum. Frontiers in Microbiology. 2024;14. 10.3389/fmicb.2023.1254882 PMC10800739 38260893

[77] Jumper J Evans R Pritzel A , et al. Highly accurate protein structure prediction with AlphaFold. Nature. 2021;596(7873):583-589. Statements and Declarations. 34265844 10.1038/s41586-021-03819-2 PMC8371605

