# Supplementary material for: Hidden in the Pangenome? Machine Learning-Driven Discovery of Antimicrobial Potential in Corynebacterium glutamicum
Source: Bioinform Biol Insights. 2026 Jun 17;20:11779322261461933. doi: 10.1177/11779322261461933 (PMC13276223; doi:10.1177/11779322261461933)
Supplement: Supplemental Material - Hidden in the Pangenome? Machine Learning-Driven Discovery of Antimicrobial Potential in Corynebacterium glutamicum [file sj-pdf-1-bbi-10.1177_11779322261461933.pdf]

Table 1. Details of the dataset of *Corynebacterium glutamicum*

**Assembly Number**	**Strain Name**	**Genome Size**	**Number of Genes**
GCA_000010225.1	R	3.4 Mb	3238
GCA_000011325.1	ATCC 13032	3.3 Mb	3099
GCA_013014885.1	ATCC 21799	3.3 Mb	3163
GCA_013046805.1	BE	3.3 Mb	3208
GCA_001447865.2	CP	3.3 Mb	3121
GCA_001518935.2	USDA-ARS-USMARC-56828	3.3 Mb	3062
GCA_001643035.1	YI	3.3 Mb	3203
GCA_001683115.1	ZL-6	3.3 Mb	3086
GCA_001687645.1	ATCC 13869	3.3 Mb	3075
GCA_017579845.1	BCA	3.1 Mb	2844
GCA_018286655.1	CGMCC1.15647	3.4 Mb	3232
GCA_018884245.1	TCCC11822	3.3 Mb	3109
GCA_001936195.1	XV	3.3 Mb	3165
GCA_019504365.1	CR101	3 Mb	2894
GCA_019551735.1	ATCC 21573	3.2 Mb	2913
GCA_000196335.1	DSM 20300 = ATCC 13032	3.3 Mb	3138
GCA_002163915.1	TQ2223	3.3 Mb	3121
GCA_002220135.1	WM001	3.3 Mb	3086
GCA_002243555.1	ATCC 14067	3.3 Mb	3222
GCA_002277975.1	C1	2.8 Mb	2712

GCA_002355675.1	AJ1511	3.3 Mb	3093
GCA_027569975.1	ATCC 13032	3.3 Mb	3053
GCA_002847405.1	*Corynebacterium glutamicum* ATCC 13032	3.3 Mb	3139
GCA_002847425.1	*Corynebacterium glutamicum* ATCC 13032	3.3 Mb	3169
GCA_029674605.1	Cg21420	3.3 Mb	3067
GCA_000382905.1	ATCC 13032 substr. K051	3.3 Mb	2992
GCA_000404145.1	SCgG1	3.4 Mb	3101
GCA_000404185.1	SCgG2	3.4 Mb	3103
GCA_000445015.1	MB001	3.1 Mb	2945
GCA_000742715.1	ATCC 21831	3.2 Mb	2932
GCA_000742735.1	AR1	3.2 Mb	2901
GCA_007430945.1	JH41 parent	3.1 Mb	2954
GCA_007431185.1	JH41	3.1 Mb	2956
GCA_007833315.1	B414	3.2 Mb	2938
GCA_007833335.1	CICC10064	3.2 Mb	2940
GCA_000828015.1	B253 (strain)	3.2 Mb	2984

Table 2. Biosynthetic gene clusters distribution and class

Assembly Number	Number of BCG per Genome	Biosynthetic	Hybrid Biosynthetic Gene	BCG Class
GCA_000010225.1	25	5	20	Terpene
GCA_000011325.1	23	4	19	Terpene
GCA_013014885.1	23	4	19	Terpene
GCA_013046805.1	26	5	21	Terpene
GCA_001447865.2	23	4	19	Terpene
GCA_001518935.2	23	4	19	Terpene
GCA_001643035.1	26	5	21	Terpene
GCA_001683115.1	23	4	19	Terpene
GCA_001687645.1	23	4	19	Terpene
GCA_017579845.1	15	3	12	Terpene
GCA_018286655.1	25	5	20	Terpene
GCA_018884245.1	23	4	19	Terpene
GCA_001936195.1	23	4	19	Terpene
GCA_019504365.1	23	4	19	Terpene
GCA_019551735.1	23	4	19	Terpene
GCA_000196335.1	23	4	19	Terpene
GCA_002163915.1	22	4	18	Terpene
GCA_002220135.1	23	4	19	Terpene

GCA_002243555.1	26	5	21	Terpene
GCA_002277975.1	14	2	12	N/A
GCA_002355675.1	23	4	19	Terpene
GCA_027569975.1	23	4	19	Terpene
GCA_002847405.1	23	4	19	Terpene
GCA_002847425.1	23	4	19	Terpene
GCA_029674605.1	23	4	19	Terpene
GCA_000382905.1	23	4	19	Terpene
GCA_000404145.1	25	5	20	Terpene
GCA_000404185.1	25	5	20	Terpene
GCA_000445015.1	23	4	19	Terpene
GCA_000742715.1	23	4	19	Terpene
GCA_000742735.1	23	4	19	Terpene
GCA_007430945.1	23	4	19	Terpene
GCA_007431185.1	23	4	19	Terpene
GCA_007833315.1	23	4	19	Terpene
GCA_007833335.1	23	4	19	Terpene
GCA_000828015.1	28	6	22	Polyketide
